# Supplementary figures and images for: A moonlighting function of a chitin polysaccharide monooxygenase, CWR-1, in Neurospora crassa allorecognition
Source: eLife. 2022 Aug 30;11:e80459. doi: 10.7554/eLife.80459 (PMC9550227; doi:10.7554/eLife.80459)

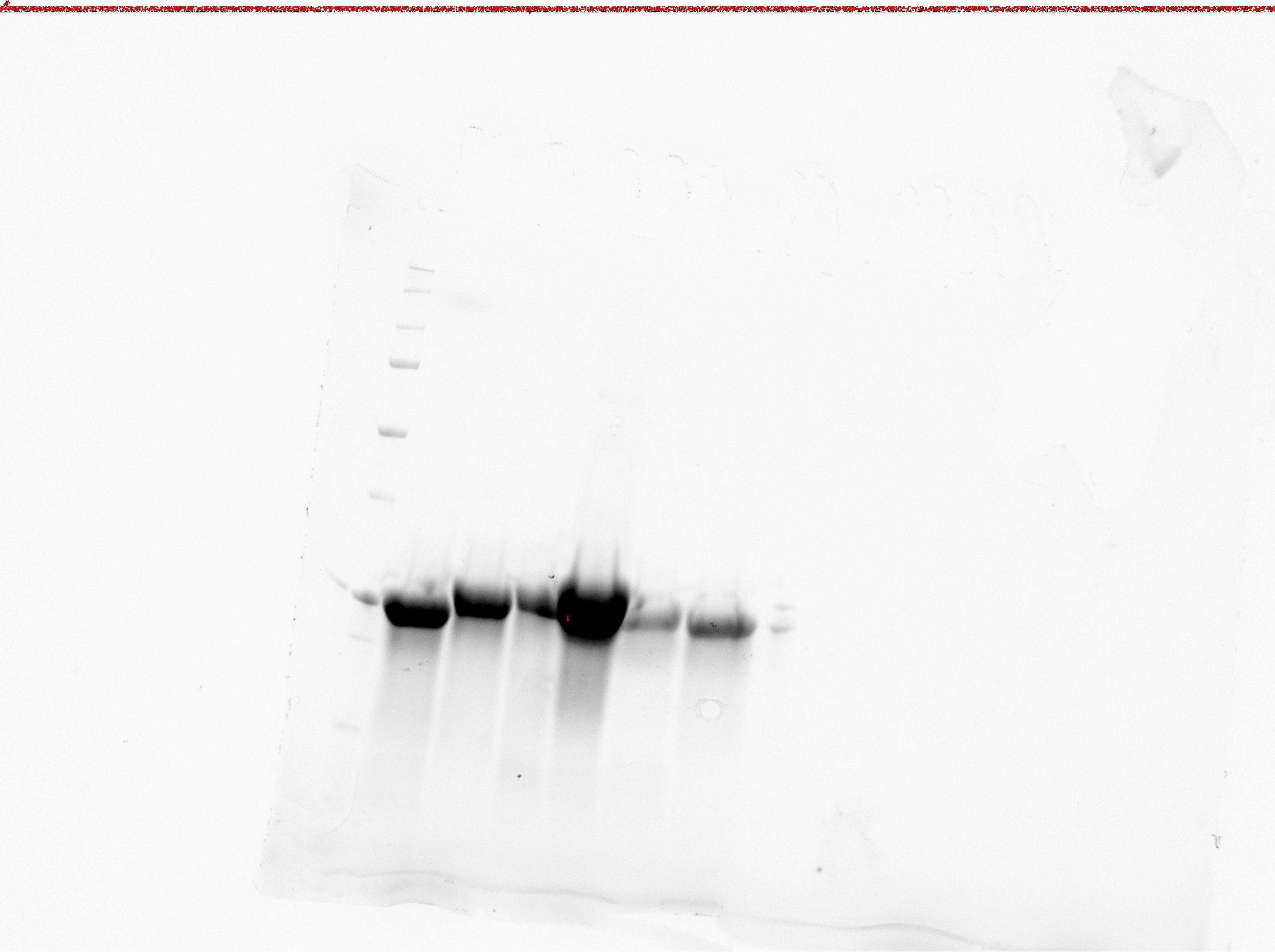

Supplement: Figure 1—figure supplement 4—source data 1. — An SDS-PAGE stain-free gel of the purified CWR-1 PMO domains from each haplogroup. The order of the lanes from left to right is: ladder (Precision Plus Protein Standards unstained Bio-Rad) HG 6, HG4, HG3, HG5, HG2, HG1. [file elife-80459-fig1-figsupp4-data1.zip › Figure 1-figure supplement 4-source data 1.tif]
